# Photopic pupil size, foveal anatomy and emmetropisation: a cross-sectional study in adult eyes

**DOI:** 10.1136/bmjophth-2026-002836

**Published:** 2026-07-16

**Authors:** Niklas Domdei, Brian Vohnsen, Siegfried Wahl, Wolf Harmening

**Affiliations:** 1 Carl Zeiss Vision International GmbH, Aalen, Germany; 2 Optics Group, School of Physics, University College Dublin, Dublin, Ireland; 3 Institut for Ophthalmic Research, Eberhard Karls Universitat Tubingen, Tubingen, Germany; 4 Department of Ophthalmology, University Hospital Bonn, Bonn, Germany

**Keywords:** Retina, Optics and Refraction, Pupil

## Abstract

**Objective:**

To investigate the relationship between photopic pupil size, refractive state and foveal anatomy in healthy adults, in the context of emmetropisation and myopia.

**Methods and analysis:**

In this cross-sectional study, pupil sizes were recorded in 80 healthy adults (28±8 years/55 women) using a custom-built high-resolution pupil tracker (0.03 mm/pixel). Daylight lamps provided two high-luminance conditions (6.5k and 10k cd/m²). Objective refraction and axial length were measured, and additionally high-resolution optical coherence tomography (OCT) was applied to record retinal volume scans of the fovea. OCT B-scans were automatically segmented for the internal limiting membrane and retinal pigment epithelium layers by the instrument algorithm. Additionally, the ellipsoid and phagosome zones defining the outer segment lengths were determined by a custom-made algorithm. Associations between photopic pupil size, refractive state, axial length and foveal metrics were analysed with correlation and multivariable regression.

**Results:**

Photopic pupil sizes at 6.5k cd/m² ranged from 1.77 to 3.38 mm (2.33±0.26 mm). Spherical equivalent of refraction ranged from −9.4 to +4.9 D (−1.24±2.29 D), and axial length ranged from 21.0 to 27.0 mm (23.9±1.2 mm). Photopic pupil size was significantly correlated with axial length and refractive state (all p≤0.01), although the explained variance was modest (R^2^ up to 0.09). Outer segment length and foveal hill distance were significantly associated with refractive state (all p<0.05).

**Conclusion:**

The association of photopic pupil size with axial length and refractive state, alongside foveal parameters being linked to refractive state but not to pupil size (exception: nasal hill height), suggests a potential interplay between foveal architecture and emmetropisation. Further investigation via longitudinal studies of children will be required. The main limitation of this study is the under-representation of high-myopes.

**WHAT IS ALREADY KNOWN ON THIS TOPIC**

- Time spent outdoors protects against myopia. A recent model suggests that photopic pupils support optimal fovealisation and emmetropisation but this has not been empirically tested in humans.

**WHAT THIS STUDY ADDS**

- By measuring photopic pupil size, eye biometry and foveal anatomy in healthy adults, this study demonstrates that photopic pupil size is significantly associated with axial length and refractive state. The comparison of foveal parameters with refractive state supports the hypothesis of a mixture between global ocular expansion and equatorial stretching during myopic eye growth.

**HOW THIS STUDY MIGHT AFFECT RESEARCH, PRACTICE OR POLICY**

- Photopic pupil size in adults may reflect aspects of the emmetropisation process and could serve as an auxiliary biomarker for myopia risk. Furthermore, it should be considered in the design and interpretation of atropine-based treatments.

## Introduction

 One of the most successful myopia prevention strategies for children is to increase the amount of time spent outdoors.^1 2^ Several explanations have been proposed for this effect, including differences in spatial frequencies,^3 4^ illumination^5 6^ and the overall light spectrum.^7 8^ A recent physiological model based on photoreceptor light capture suggests that especially the photopic pupil size plays a crucial role in the fovealisation process and emmetropisation of the eye.^9^

Fovealisation describes the development of the fovea, the central region of the retina, which sets in during gestation and finishes at an age of about 7 years.^10 11^ A series of coordinated developmental events shape the foveal pit, notably the lateral displacement of post-receptoral neurons. Thus, the foveola, defined as the innermost 0.6° of the fovea,^12^ lacks overlying neural layers, permitting light to pass to the photoreceptors without undesired scattering. At the same time, cone photoreceptors shift inward to establish a densely packed mosaic at their smallest diameters anywhere in the retina,^13^ and their outer segments elongate.^10^

According to the angular spectrum model proposed by Vohnsen,^9^ a small photopic pupil provides the optical conditions required to minimise photoreceptor outer segment leakage and crosstalk. By restricting the angular vergence of light at the retina, a sufficiently small pupil (≈3 mm or less during childhood) is predicted to preserve directional light capture and support the orderly migration and dense packing of cones towards the foveal centre. In contrast, a larger (mesopic) pupil increases angular spread, potentially degrading the fidelity of retinal signals needed to detect small defocus changes.^14^ Within this framework, impaired suppression of crosstalk during development could weaken the emmetropisation feedback loop^15^ and contribute to excessive elongation both in the central and peripheral retina.

Leakage of light from outer segments has previously been used to explain the Stiles-Crawford effect of the first kind,^16 17^ and reduced directionality with increasing axial length has been described as a geometric scaling effect.^18^ Extending this reasoning, persistently larger pupils under indoor conditions will increase angular spread and aberrational blur, potentially masking the eye’s refractive state.^19^,^21^ Studies examining associations between pupil size and refractive error have yielded mixed results: larger cohort studies (n>100) report significant correlations with mesopic pupil size,^22^,^25^ whereas smaller studies (n<100) generally do not.^2126^,^29^

Notably, most previous studies did not assess pupil size under daylight-like photopic conditions and did not consider detailed foveal morphology. If the proposed model holds, photopic pupil size should relate not only to axial length and refractive state but potentially also to structural markers of fovealisation. Therefore, we measured minimal photopic pupil size under high-luminance conditions and combined these data with high-resolution optical coherence tomography (OCT) imaging of the fovea to evaluate associations between pupil size, axial length, refractive state and foveal anatomy.

## Materials and methods

### Participants

A total of 80 participants (55 women/25 men), students or employees of the University or University Eye Hospital of Bonn, were recruited (age range/median: 18–51/26.5 years). Participants had to be eye healthy, with no cases of refractive surgery and no known neurological conditions.

### Eye measurements

Both eyes of all participants were examined by the following protocol: biometry of the eye was assessed by non-cycloplegic objective refraction (TonoRef 3; Nidek/Oculus Optikgeräte GmbH, Wetzlar, Germany) and Swept-Source OCT-based axial length measurement (IOLMaster 700; Carl Zeiss Meditec, Jena, Germany). Cycloplegia was not used to keep the study protocol non-invasive based on the fact that the also recorded axial length provides redundancy and as it was shown that non-cycloplegic objective refraction is sufficiently accurate.^30^ Eye dominance was found with the Miles test: participants were asked to form a triangle with the hands at arm’s length and look through the triangle with both eyes open at a distant target three times. The eye seeing through the triangle at least two times was reported as the dominant eye.

High-resolution foveal volume scans with an axial resolution of about 2 µm in air were acquired using a prototype OCT device (High-res OCT; Heidelberg Engineering GmbH, Heidelberg, Germany). The imaging field was centred on the fovea, covering 15° horizontal and 2.5° vertical and containing 128 B-scans each with 768 A-scans (about 6 µm lateral spacing). Conversion from degree scanning angle into retinal distance units was achieved by calculating the retinal magnification factor of each eye based on corneal radius, anterior chamber depth and axial length.^31^

**Figure 1:**
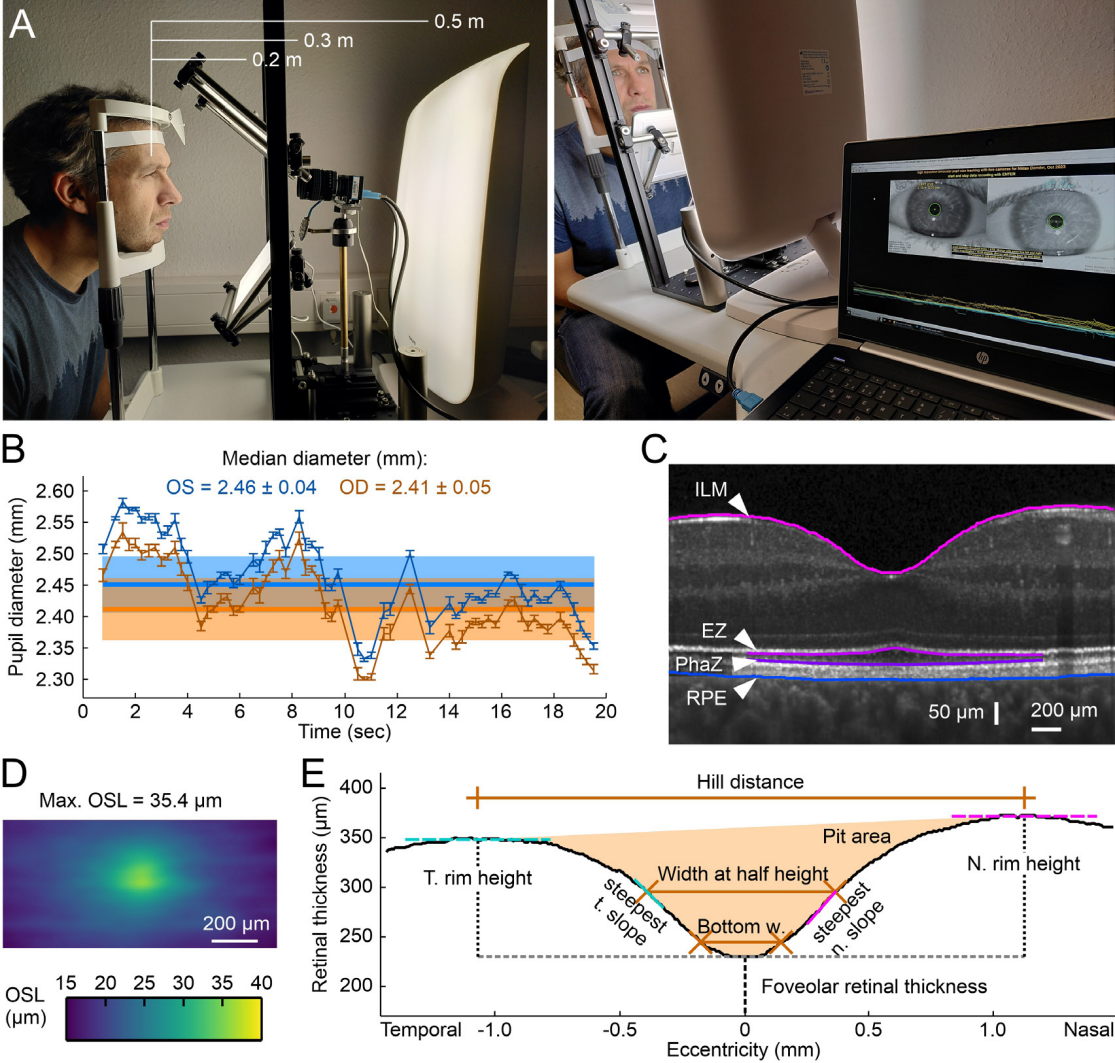
Overview of the main measurements and analysis pipeline. (**A**) The custom binocular pupil tracker with daylight illumination (TL30 and TL90, each 10k lux) during photopic pupil measurement in one of the authors of the study. (**B**) Exemplary pupil-tracker recording used to determine the photopic pupil size after eye blink removals. Error bars indicate the SD from pooling the 125 Hz recording into 250 ms bins. The final pupil size was given as the median diameter from a 10–20 s video recording. (**C**) Segmentation of outer segment length (OSL) in a high-resolution OCT B-scan, determined as the distance between the ellipsoid zone (EZ) and phagosome zone (PhaZ) enables the generation of a foveolar OSL map (shown in **D**). The internal limiting membrane (ILM) and retinal pigment epithelium (RPE) were segmented by the intrinsic Heidelberg Engineering algorithm. All OCT scans, including their respective segmentation, were rotated by balancing the RPE layer on both sides of the B-scan. (**E**) The following parameters describing the fovea were extracted: Nasal and temporal height of the foveal rim, hill distance, width at half hill height and 10% height (=bottom width), steepest nasal/temporal slope, foveal pit area and central retinal thickness. OCT, optical coherence tomography.

The smallest photopic pupil diameter was measured with a custom-built binocular pupil tracker^32^ with a spatial resolution of 0.03 mm/pixel (working distance: 0.3 m) and temporal resolution of 125 Hz. Head position was immobilised by a head and chin rest. The pupil tracking cameras were adjusted to achieve optimal focus of the pupil. Controlled daylight scenarios with 6.5k and 10k cd/m² were realised by three daylight lamps each providing an illuminance of 10k lux: Two smaller ones (TL30; Beurer Europe GmbH, Ulm, Germany) at about 0.2 m distance in the periphery and a large one (TL90) 0.5 m central in the line of sight (figure 1A). The spectra were measured with a spectroradiometer (Spectra Scan PR655, Photo Research) (see online supplemental figure 1). To avoid obscuring the pupil size, participants did not wear any corrections like spectacles or contact lenses. Pupil size was measured for 10–20 s, depending on the participant’s compliance. Recording started after a short adaptation period to the current illumination.

### Data processing and statistics

The pupil tracker’s data points recorded with 125 Hz were pooled into 250 ms bins. After removal of eye blinks and noisy data points with high fluctuations due to uncertain pupil tracking, caused by incomplete eye blinks, squinting or eyelashes, photopic pupil size was determined as the median from the full measured sequence (figure 1B). To obtain foveolar outer segment length (OSL) from OCT scans, a custom segmentation algorithm was used (figure 1C).^33^ For segmentation of the internal limiting membrane and retinal pigment epithelium, the device intrinsic Heidelberg Engineering algorithm was used (figure 1D) on which the other foveal parameters assessed in this study (figure 1E) are based. To achieve non-biased height measurements for the final analysis, OCT scans including their respective segmentation were rotated by balancing the retinal pigment epithelium layer on both sides of the B-scans. To maintain statistical independence, results from only the dominant eyes are presented throughout this study (except for within participant comparisons).

All numerical and statistical analyses were performed in Matlab (2022a; The Mathworks, Natick, Massachusetts, USA). The foveal pit area was calculated as numerical integration (Matlab: trapz). Correlations were calculated based on the F-test (Matlab: regress) and CIs computed using the Matlab functions: ‘fitlm’ and ‘predict’. For the multi-regression model ‘fitlm’ was applied.

### Patient and public involvement

The participants were not involved in the design, conduct, reporting, or dissemination plans of our research.

## Results

The data of one participant were excluded from the foveal pit shape analysis because of foveal hypoplasia. In two more participants, the custom segmentation algorithm for OSL determination failed. The respective number of eyes analysed is stated in the figures.

**Figure 2:**
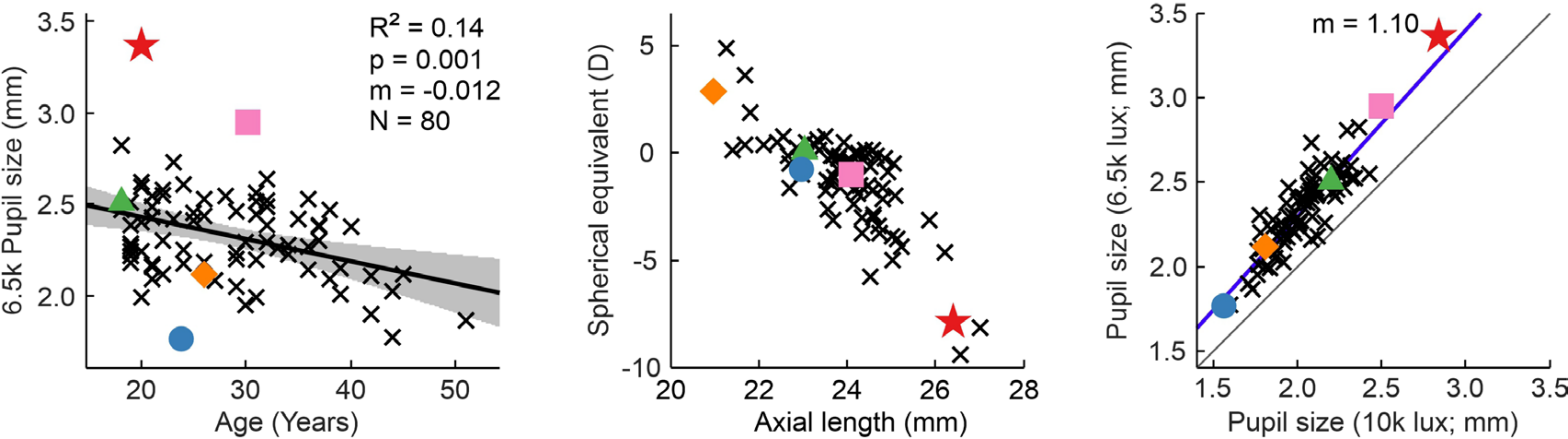
Biometry data of dominant eyes in the study cohort (n=80). The photopic pupil size at 6.5k cd/m² was correlated with age, yielding a slope of −0.012 mm/year. The scatter plot showing the distribution of spherical equivalent and axial length reveals the typical 3 D/mm relationship. On average, the pupil size differed by 0.3 mm between the two illumination scenarios, showing a slope of m=1.1 across all eyes examined. The five markers highlight results for a subset of participants to ease comparison.

The 80 participants of this study were between 18 and 51 years old (28±8 years; mean±SD) on the date of data recording. A significant correlation between photopic pupil size and age was observed (R^2^=0.14; p=0.001), with a slope of −0.012 mm per year (figure 2). The analysis highlights findings with markers for five participants, selected by their pupil size, in figures2^4^. The average refractive error was −0.95±2.20 D in sphere (min: −8.00 D; max: 5.25 D) and −0.57±0.56 D in cylinder (min: −2.75 D; max: 0) or −1.24±2.29 D expressed as spherical equivalent of refraction (SER; min: −9.38 D; max: 4.88 D). Axial length was on average 23.9±1.2 mm with a range between 21.0 mm and 27.0 mm. The distribution of observed pupil sizes followed nearly a 1:1 ratio (m=1.1) for the two different illumination scenarios (6.5k and 10k cd/m²), with an average offset of about 0.3 mm. For 6.5k cd/m², the observed pupil size was between 1.77 and 3.38 mm (2.33±0.26 mm) and for 10k cd/m² between 1.57 and 2.84 mm (2.03±0.21 mm). To remove redundancy, only the 6.5k cd/m² data are presented in the following. The 10k data produced similar correlations (see online supplemental figure 2).

**Figure 3:**
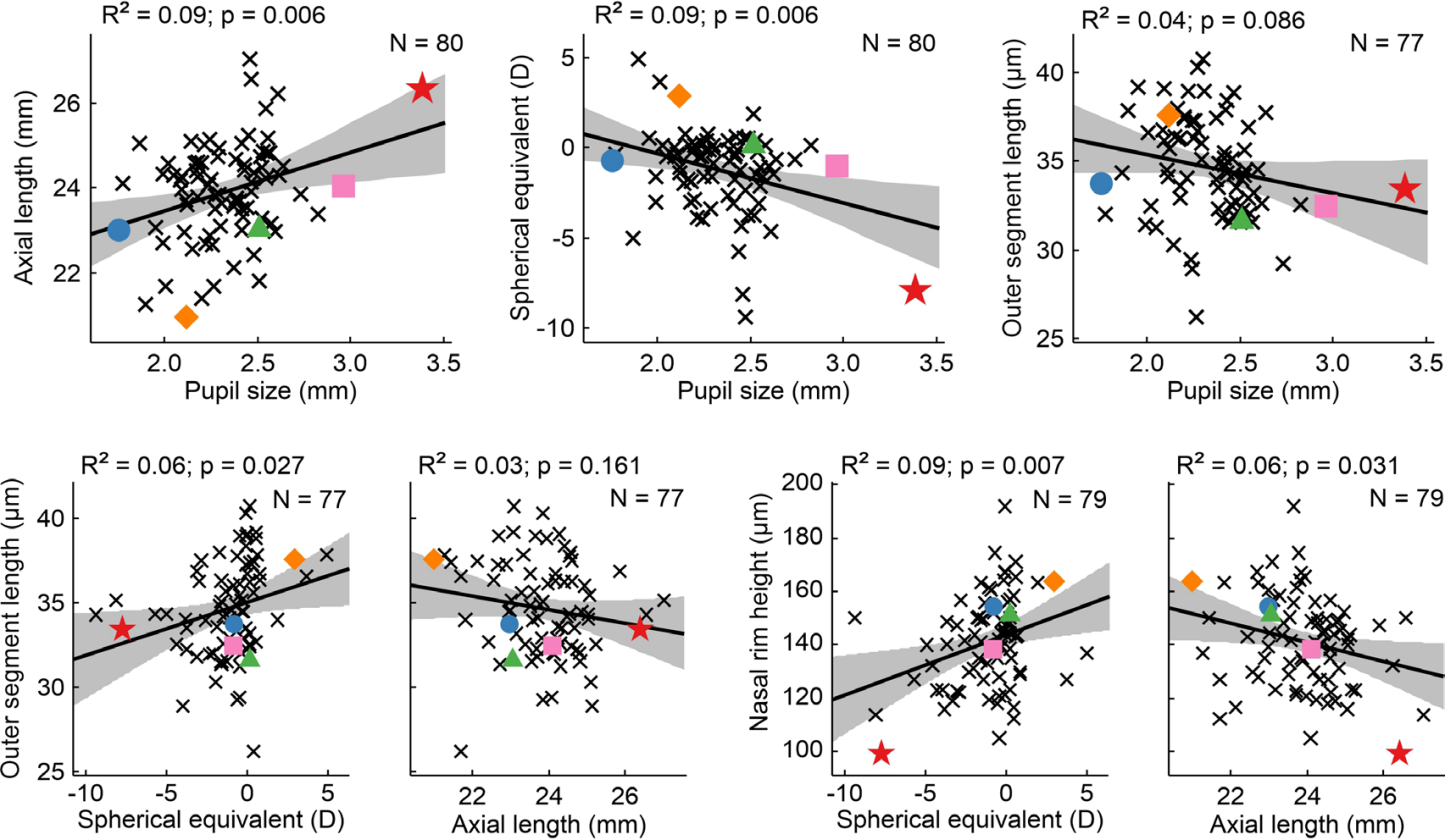
Correlations between the photopic pupil size (with 6.5k cd/m²) and the eye’s refractive state as well as selected foveal anatomy parameters.

**Figure 4:**
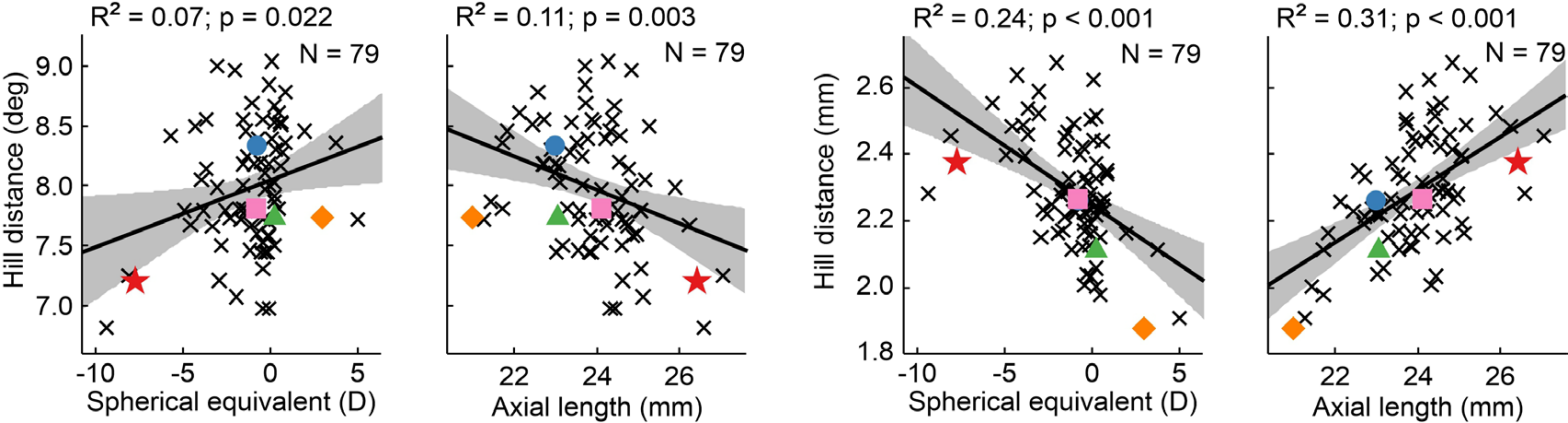
Correlations of foveal hill distance in angular and metric units with SER and axial length. SER, spherical equivalent of refraction.

Pupil size was significantly correlated with axial length (R^2^=0.09; p<0.01) and SER (R^2^=0.09; p<0.01) (see figure 3, top row), or sphere (data not shown). When adjusting pupil size for age, this did not change the observed correlations between pupil size and the measured parameters, for example, SER (R^2^=0.08; p=0.01). For the foveal parameters, a significant correlation between pupil size and nasal hill height was found (R^2^=0.06; p<0.05). All other quantified parameters (OSL, hill distance, width at half hill height and bottom, steepest nasal/temporal slope, foveal pit area and central retinal thickness), were not significantly correlated with pupil size (all R² <= 0.04). Photopic pupil sizes were not different between female and male participants (p=0.06; Mann-Whitney U test; see online supplemental figure 3), and highly correlated between dominant and non-dominant eyes (R^2^= 0.92).

Subsequently, the relationship between foveal characteristics and emmetropisation was investigated (see figure 3, bottom row). OSL was significantly correlated with SER (R^2^=0.06; p<0.05), but not with axial length (R^2^=0.03). Also, an increased nasal hill was observed in shorter respectively emmetropic/hyperopic eyes, yielding a significant correlation between nasal rim height (but not temporal rim height) and SER, as well as axial length (R^2^=0.09 and 0.06; p<0.01 and < 0.05, respectively).

Additionally, the foveal shape was analysed by comparing its angular and metric dimensions to better understand myopic eye growth (see figure 4). Overall, the hill distance in degree scanning angle was significant and even more pronounced in millimetres. It was significantly correlated with SER and axial length (R^2^=0.07, 0.24, 0.11 and 0.31; p<0.05, <0.001, <0.01 and <0.001, respectively). The linear regression slope was negative for the relationship between axial length and angular hill distance, and positive for the relationship between axial length and metric hill distance.

**Figure 5:**
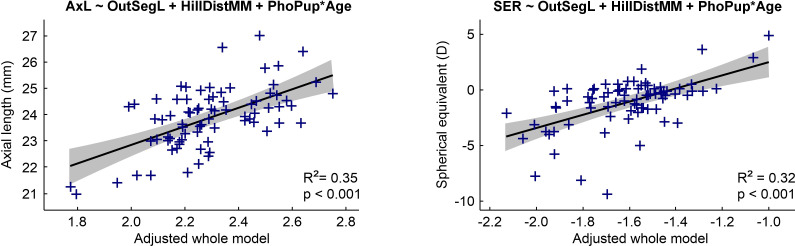
Multilinear regression models predicting axial length and SER based on foveal anatomy and an age-corrected photopic pupil size. SER, spherical equivalent of refraction.

Combining these findings, a multi linear regression model was used to investigate the prediction capability for successful emmetropisation defined by axial length growth and SER based on foveal parameters (OSL and hill distance) in combination with an age corrected photopic pupil size (see figure 5). This model (AxL | SER~OSL + HillDistance+PhotopicPupil*Age) yielded an overall R^2^ value of xL = 0.35 and SER = 0.32, respectively. Removing the photopic pupil size from this model reduced the R^2^ to 0.32 or 0.29.

## Discussion

This study tested the hypothesis derived from the angular spectrum model that photopic pupil size is linked to foveal architecture and emmetropisation. In healthy adults, photopic pupil size was significantly associated with axial length and refractive state, although the explained variance was modest (R^2^≈0.09). Most structural markers of fovealisation, especially OSL, were not associated with pupil size. These findings suggest that while photopic pupil size may contribute to refractive development, its direct influence on foveal morphology appears limited.

Before interpreting these associations, it is important to confirm that our photopic pupil measurements are consistent with established normative data. The average photopic pupil size of 2.33±0.26 mm at 6.5k cd/m² closely matches previous reports in comparable bright environments.^34 35^ It also agrees with the estimate of 2.19 mm derived from the unified formula of Watson and Yellott for a 28-year-old observer at this luminance.^36^ The age-related decrease in pupil size observed here (−0.012 mm/year) aligns well with earlier findings (−0.015 mm/year)^27^ and further supports the validity of our measurements.

The angular spectrum model proposes that a sufficiently small pupil during childhood restricts angular vergence within the eye, minimising photoreceptor leakage and crosstalk,^9^ which is consistent with both a non-guided and a waveguide-based analyses of cone photoreceptors.^37^ Thus, light-evoked retinal activity may promote the observed cone packing and outer segment elongation during foveal development.^38^ Under this framework, one would expect that adult photopic pupil size reflects early optical conditions and might therefore be associated with structural markers of fovealisation. In our cross-sectional adult cohort, OSL, used as a surrogate for central cone density,^33^ showed only a non-significant tendency towards shorter values in eyes with larger photopic pupils. Apart from a weak association with nasal hill height (R^2^=0.06), no systematic relationship between pupil size and foveal morphology was observed. These findings suggest that photopic pupil size does not strongly shape foveal structure in the mature eye. Given that foveal maturation follows a tightly regulated developmental trajectory,^39^ structural outcomes may be relatively resistant to variation in optical boundary conditions alone.

In contrast, photopic pupil size was significantly associated with axial length and SER. Although the effect size was modest (R^2^=0.09), this relationship was robust. Previous studies examining pupil size and refractive error have reported mixed results, with significant associations typically emerging only in large cohorts.^21^,^29^ Many of these studies assessed mesopic rather than photopic pupil size and relied on group comparisons rather than continuous analyses, potentially obscuring subtle but systematic relationships. The present findings indicate that under high-luminance conditions, a small but consistent association exists between pupil size and refractive development, as suggested by the angular spectrum model. The magnitude of this effect appears small, however. Inclusion of age-corrected photopic pupil size in multivariable regression models only slightly improved the prediction of SER (R^2^ from 0.29 to 0.32). Thus, pupil size alone cannot account for substantial variance in refractive outcome but may represent one contributing factor within a multifactorial growth regulation process.

We considered several potential confounders in our study. No significant gender differences in photopic pupil size were observed in this cohort (see online supplemental figure 3). Although iris pigmentation has been suggested to influence light sensitivity, previous outdoor measurements did not demonstrate systematic differences between light and dark irises.^34^ Although body height is not related to pupil size,^40^ it is known to correlate with axial length in adults^41^ and axial length growth in children.^42^ But it is unlikely that it affected the observed associations here, as similar relationships were found for both axial length and SER. Accommodation-induced pupil changes are also unlikely to have influenced the results given the high luminance levels^43^ and the consistency of findings across illumination conditions.

Interestingly, structural foveal parameters showed stronger associations with refractive state than pupil size did. Foveal hill distance exhibited a substantial correlation with axial length (R^2^ up to 0.31 in metric units). OSL, as a surrogate for central cone density, was modestly associated with refractive state, consistent with adaptive optics studies reporting reduced cone density in longer eyes.^44 45^ Moreover, the finding that angular hill distance decreases while metric hill distance increases with axial length supports the concept of combined global expansion and equatorial stretching during myopic eye growth.^44^ A previous study testing foveal shape parameters in the context of myopia failed to confirm this observation, reporting no significant correlation between axial length and foveal width.^46^ However, the measurement used by Breher *et al* to determine foveal width corresponds to the full width half maximum measurement in this study,^47^ which shows no significant relationship with axial length or SER, too. Moreover, a horizontal asymmetry was observed here with the temporal hill height being stable while the nasal hill being significantly positively correlated with SER (or negatively with axial length). Such horizontal asymmetry in relation to the eye’s refractive state was recently also reported from peripheral refractive measurements.^48^

Whether or not the fovea plays an important role in emmetropisation is still an ongoing discussion.^49^ There is strong evidence that visual input from the fovea is not strictly required for normal refractive development, whereas near-peripheral retinal signals appear to be critical for emmetropisation. For example, in rhesus monkeys with ablated foveas, it was observed that the peripheral retina in isolation can support emmetropisation.^50^ Additionally, electrophysiological and behavioural studies in humans further suggest that the near periphery (6–12° eccentricity), is a key region for blur detection and defocus-driven growth signals.^51 52^ In contrast, individuals with albinism, who typically exhibit foveal hypoplasia, show a wide distribution of refractive errors ranging from high myopia to high hyperopia and very few emmetropic eyes.^53^,^55^ However, albinism is also associated with other retinal and anterior segment abnormalities. In non-albinotic foveal hypoplasia, refractive outcome is largely independent of the degree of foveal underdevelopment indicating that foveal maldevelopment in isolation does not substantially disrupt the emmetropisation process.^56^ In accordance with the angular spectrum model, the reason for emmetropisation failure in albinism could be the translucent iris and increased intraocular straylight due to thinning of the iris pigment epithelium and altered light transmission within the eye.^57 58^ Similarly, aniridia and PAX6-related anterior segment dysgenesis, characterised by a large and poorly regulated pupil aperture, are associated with a high prevalence of myopia.^59 60^

The potential role of pupil size remains relevant in clinical contexts of myopia control. Orthokeratology has been associated with reduced photopic pupil size and slower axial elongation,^61^ which would align with the concept that reduced angular spread may favour more stable growth regulation. Conversely, peripheral defocus spectacle lenses have shown greater efficacy in children with larger pupils,^62^ where induced peripheral aberrations may be more pronounced. Atropine treatment, while increasing pupil size, exerts its primary effects through atropine being a non-specific muscarinic cholinergic antagonist and thus interacting on several layers with the retina’s and scleral’s biochemistry controlling axial length growth.^15 63^ In this context, pupil dilation may represent an optical counteracting factor rather than a therapeutic mechanism, and combined optical interventions could potentially enhance treatment efficacy.

In summary, photopic pupil size does not appear to be a dominant determinant of adult foveal morphology, likely because foveal maturation is strongly genetically regulated. Nevertheless, pupil size is associated with refractive state, indicating a measurable, although modest, role in emmetropisation. Structural retinal remodelling shows a stronger relationship with axial elongation than pupil size does, suggesting that optical boundary conditions may modulate, but do not dictate, the trajectory of eye growth. Longitudinal studies in children are required to determine whether photopic pupil size exerts a stronger influence during the active phase of foveal development and emmetropisation, whether age-dependent changes in photoreceptor density are impacted by the pupil size and how peripheral retinal changes contribute to the emmetropisation process in the context of angular confinement of light.

## Supplementary material

10.1136/bmjophth-2026-002836 online supplemental figure 1

10.1136/bmjophth-2026-002836 online supplemental figure 2

10.1136/bmjophth-2026-002836 online supplemental figure 3

10.1136/bmjophth-2026-002836 online supplemental file 1

## Data Availability

Data are available upon reasonable request.
