# Supplementary figures and images for: Photopic pupil size, foveal anatomy and emmetropisation: a cross-sectional study in adult eyes
Source: BMJ Open Ophthalmol. 2026 Jul 16;11(3):e002836. doi: 10.1136/bmjophth-2026-002836 (PMC13384153; doi:10.1136/bmjophth-2026-002836)

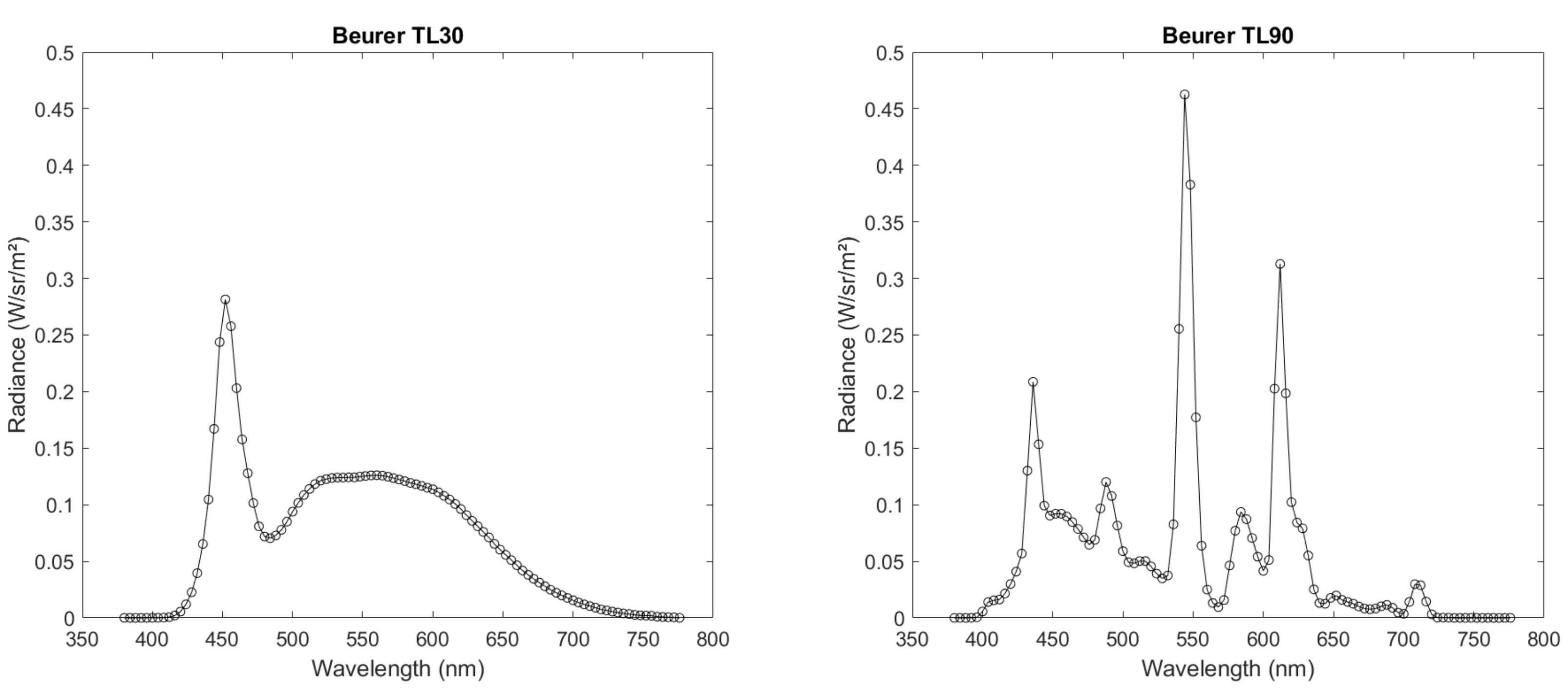

Supplement: online supplemental figure 1 [file bmjophth-11-3-s001.jpg]

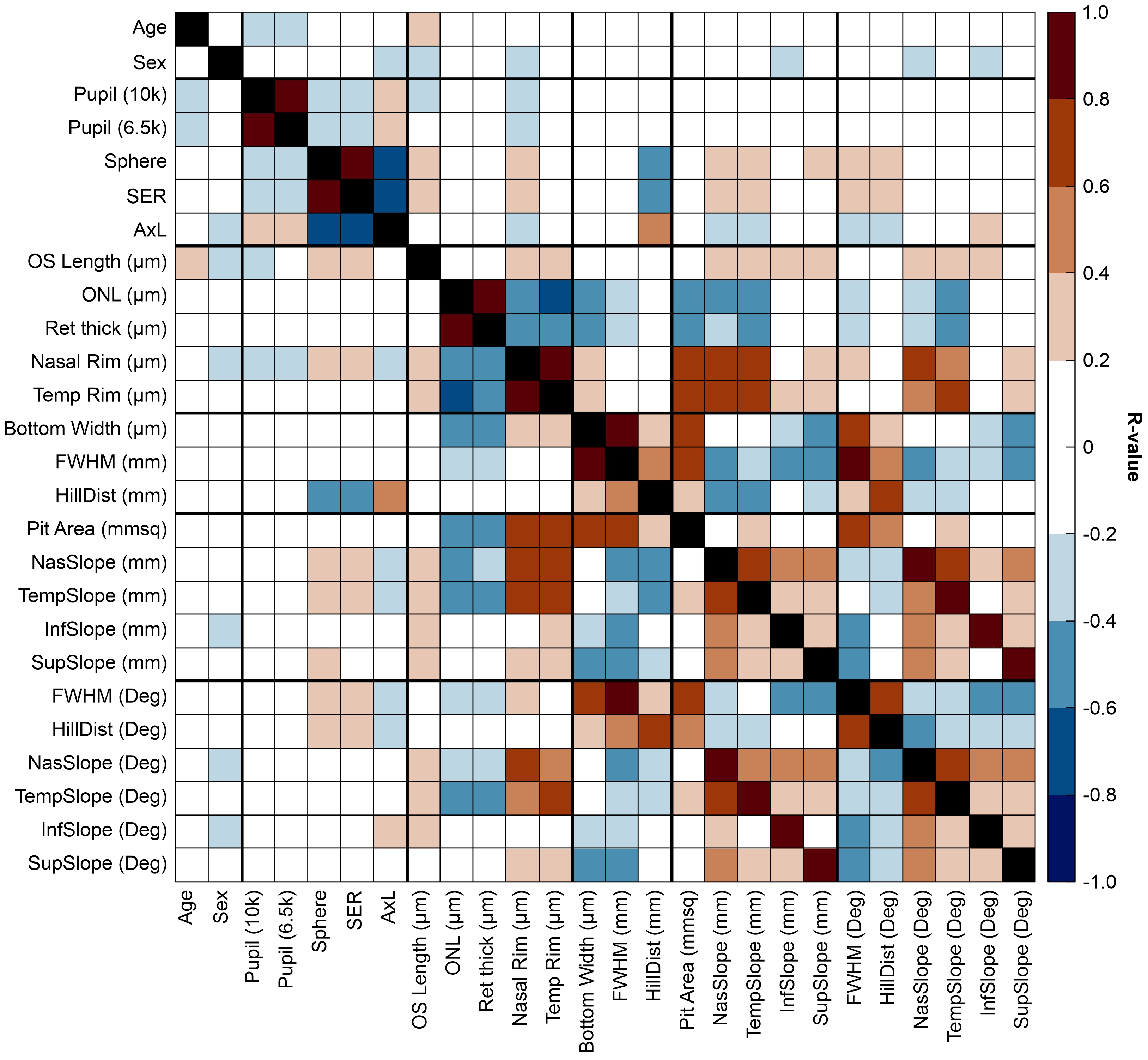

Supplement: online supplemental figure 2 [file bmjophth-11-3-s002.jpg]

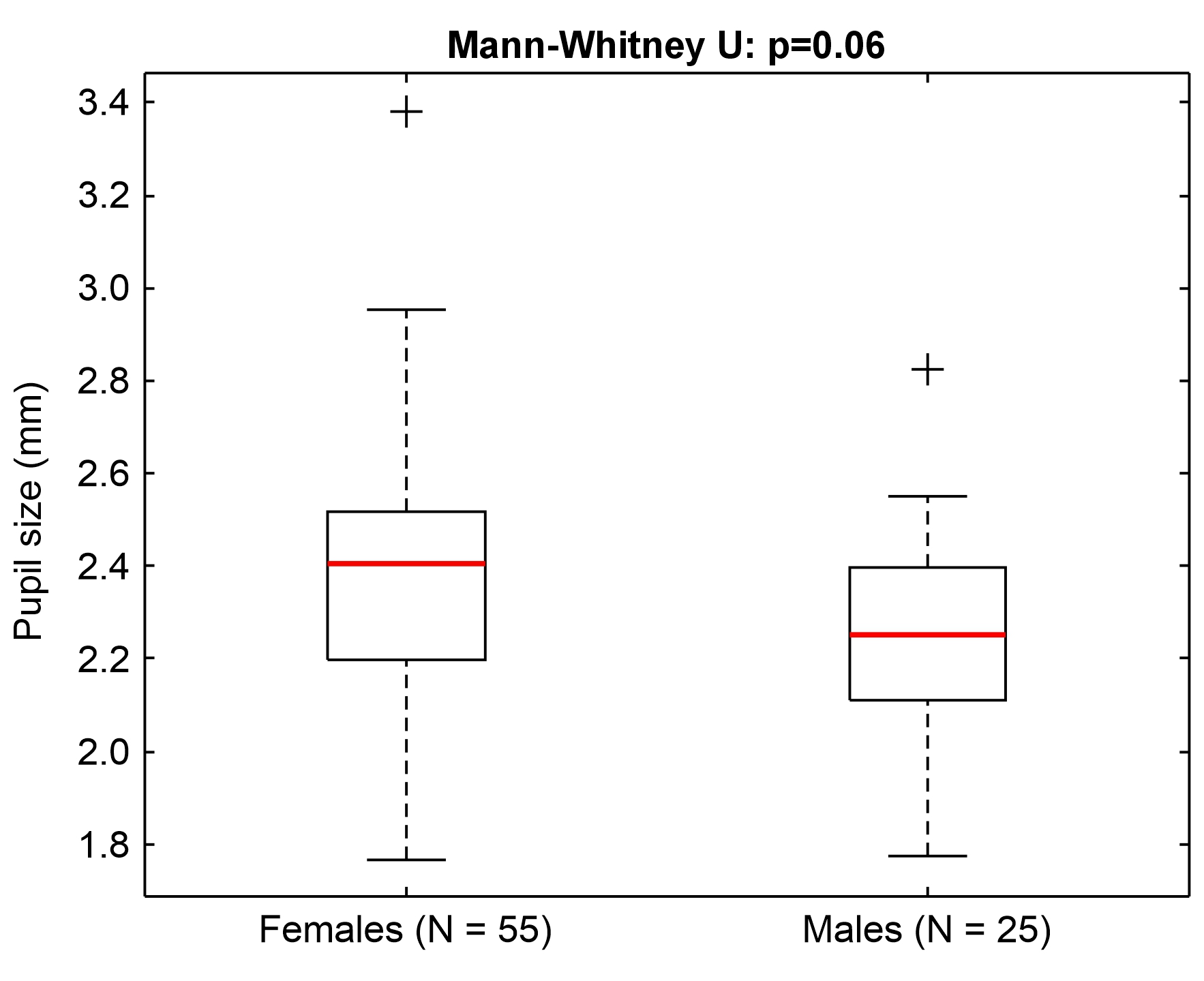

Supplement: online supplemental figure 3 [file bmjophth-11-3-s003.jpg]
