# Supplementary material for: Photopic pupil size, foveal anatomy and emmetropisation: a cross-sectional study in adult eyes
Source: BMJ Open Ophthalmol. 2026 Jul 16;11(3):e002836. doi: 10.1136/bmjophth-2026-002836 (PMC13384153; doi:10.1136/bmjophth-2026-002836)
Supplement: online supplemental file 1 [file bmjophth-11-3-s004.pdf]

Supplementary Figure legends:

Supplementary Figure 1: Spectra of daylight lamps used in this study measured with Spectra Scan PR655

Supplementary Figure 2: Correlation matrix of all parameters analyzed in this study.

Supplementary Figure 3: Statistical comparison of pupil size in relation to the participant's gender. No significant difference was found between female (N = 55) and male (N = 25) photopic pupil size.
